# LTA and FAU-X Iron-Enriched Zeolites: Use for Phosphate Removal from Aqueous Medium

**DOI:** 10.3390/ma15155418

**Published:** 2022-08-05

**Authors:** Diana Guaya, Hernán Cobos, Jhulissa Camacho, Carmen Milena López, César Valderrama, José Luis Cortina

**Affiliations:** 1 Department of Chemistry, Universidad Técnica Particular de Loja, Loja 100107, Ecuador; 2 Department of Chemical Engineering, Polytechnic University of Catalonia–BarcelonaTech (UPC), 08019 Barcelona, Spain; 3 Barcelona Research Center for Multiscale Science and Engineering, 08930 Barcelona, Spain

**Keywords:** Linde type A, faujasite X, iron (oxy)hydroxides, phosphate, adsorption, complexation, equilibrium kinetics

## Abstract

Hydrothermally synthesized Linde type A (LTA) and faujasite X (FAU-X) zeolites are low-cost and environmentally benign inorganic carriers for environmental applications. In this study, (oxy)hydroxides were incorporated onto LTA and FAU-X zeolites to promote the phosphate adsorption. The performance of LTA-Fe and FAU-X-Fe was evaluated through batch adsorption assays. A complete evaluation was performed to recover phosphate from synthetic wastewater. The effect of pH, concentration, equilibrium, and kinetic parameters on phosphate adsorption and its further reuse in sorption–desorption cycles were evaluated. LTA-Fe and FAU-X-Fe are effective for adsorption of phosphate at neutral (e.g., pH 7.0 ± 0.2) and in a broad range of phosphate concentrations. Higher ratios of adsorption capacities were obtained by synthetic zeolites enriched with iron in comparison to their parent forms. The phosphate adsorption occurred through hydrogen bonding and complexation reactions between protonated iron hydroxyl groups and phosphate anions. The phosphate monolayer adsorption was followed by diffusion through the internal pores and 80% of the equilibrium adsorption was reached within 50 min. The LTA-Fe and FAU-X-Fe can be used for phosphate recovery from wastewater treatment plants. The use of LTA-Fe and FAU-X-Fe in a tertiary wastewater treatment stage could allow to reduce the phosphate–phosphorous content, reaching the regulatory levels (equal 1 mg L^−1^ total phosphorous). The phosphate adsorption using LTA-Fe and FAU-X-Fe does not require pH adjustment, and it is endothermic. The reusability of both iron zeolites is limited, and they can be finally disposed for soil amendment applications.

## 1. Introduction

Phosphate removal from water bodies has become an important issue to control eutrophication. Eutrophication is a serious environmental problem due to natural and anthropogenic sources. However, the high phosphate contents in municipal effluents and agricultural runoff [1] are the main sources in aqueous bodies. Phosphate in the inorganic phosphorus form is the main species contained in domestic sewage from detergents and household wastes [2]. Nowadays, the problem has become even worse due to the pandemic, wherein cleaning products have been used massively.

Thus, the phosphate recovery from municipal wastewaters could become an opportunity for new phosphorous sources worldwide. The scarcity of phosphorus and not-homogeneous geographical distribution is a serious concern for agrarian economies [3]. The European Union (EU) included the phosphatic rock in the list of Critical Raw Materials [4], so its supply will be delimited by geopolitical interests and it will have market fluctuations. Conventionally, some technologies have been used for phosphate removal from wastewater streams, such as ion exchange, biological process, and chemical precipitation [5]. However, for medium- and small-sized wastewater treatment plants (WWTP), the adsorption-based solutions seem to be the most attractive techniques due to their low cost, high efficiency, and easy operation. Thus, the development of environmentally friendly phosphate adsorbents (“phosphate carriers”) is necessary, from richest side streams to more diluted main streams [6].

Several types of absorbents have been developed for phosphate recovery from wastewater. There are some low-cost adsorbents that have been researched for phosphate removal from industrial wastes [7]. However, the toxic components (e.g., heavy metals) present in its composition represent a risk for further disposal due to the leaching of these hazardous compounds from the adsorbents to the soil. Polymeric anion exchangers (e.g., resins and fibers) have been widely used for phosphate removal with higher advantages (e.g., mechanical strength, selectivity, regeneration, operation in continuous mode) in comparison to other materials [8]. However, the weakness of polymeric adsorbents is the final disposal due to its lifespan. Hence, the use of inorganic minerals (e.g., clays, zeolites) [6] is advantageous. Since their chemical composition and non-harmful nature are friendly with the environment, inorganic materials are preferred for phosphate removal and further application as soil amendment material.

Zeolites are porous hydrated crystalline aluminum–silicate materials with a framework structure containing water and typically alkaline cations. Zeolitic materials of nano size are attractive materials for the removal of pollutants due to their high surface area and higher adsorption capacities. Zeolites are characterized by a permanent negative-charged and high surface, being excellent cationic adsorbents. Particularly, the phosphate (oxyanionic specie) uptake by zeolites is enhanced by supporting metallic (oxy)hydroxides (e.g., aluminum, iron), avoiding the problems of aggregation of hydroxide particles [9]. Natural zeolites, as metallic (oxy)hydroxides support, have been used for phosphate recycle as a slow nutrient-releaser, improving the biomass production and the plant water content of clay soils [10]. Synthetic zeolites have also been obtained from wastes (coal and incineration fly ash) for phosphate removal [11]. However, the mobility of toxic elements (e.g., Pb, Hg, Ni, As, and Se) may suppose a problem for further soil application.

Zeolites synthetized from commercial sources allowed the formation of pure phases and in nanometer particle sizes that cannot be found naturally. Particularly, Linde type A (LTA) and faujasite type X (FAU-X) are commercially important zeolites produced and used as adsorbents. LTA and FAU-X type zeolites contain sodalite cages as building units, linked by double four-rings (D4Rs) forming α cages and by double six-rings (D6Rs), respectively [12]. Several methods have been developed in order to produce synthetic zeolites, such as hydrothermal method, alkali-fusion method, sol–gel method, and alkali-leaching method. However, the selection of the method depends on which zeolite will be produced [13]. The hydrothermal synthesis method is conventionally preferred because it does not require specialized equipment, and water is used as crucial solvent. Thus, the use of a sealed vessel (e.g., polypropylene) is required to operate at low temperatures. It is a direct and cheaper method of synthesis in comparison to the aforementioned. Some additional advantages have been attributed to the hydrothermal method, such as low energy consumption, high reactivity, easy maintenance, and metastable phases formed, as well as a unique condensed phase obtained [13].

The obtaining of LTA and FAU-X type zeolites by the hydrothermal method is the primary route of synthesis, which is a well-known procedure that is non-expensive and non-toxic. In comparison, the solvothermal method, where the preparation of zeolites is performed using an organic structure directing agent (SDA, organic templates), is disadvantageous due to its harmful effect and because they are removed at high temperatures (>400 °C) [14,15]. Additionally, the importance of the high purity of the starting materials used in the hydrothermal synthesis method is also well known. Then, the crystallization process is selective for the crystals grown, rejecting impurities from their structure [16]. Thus, several environmental applications can be evaluated using hydrothermally synthetized LTA and FAU-X zeolites.

Zeolites, as excellent cationic exchangers with low affinity for phosphate adsorption [9], require the incorporation of metal species (e.g., Fe^3+^) to promote the formation of iron (oxy)hydroxides that are conventionally used for phosphate removal. The formation of iron (oxy)hydroxides groups in the surface inorganic templates (e.g., clay and zeolites) become a convenient method to control the aggregation and small particle size of iron particles [6]. The incorporation of Fe^3+^ onto the zeolite surfaces under basic conditions allowed the effective formation of hydrated iron hydroxide particles as the main modification mechanism [1]. The development of this type of adsorbent material is promissory since it contains iron, which is an elemental micronutrient for plants. Based on our previous studies, the phosphate-loaded Fe-adsorbents could be used as slow-release fertilizers, where inorganic phosphate could be released under the controlled root mechanisms [17]. The application of resulting exhausted adsorbents in agriculture and in agronomical applications could be an alternative for final disposal since it is a critical aspect. Therefore, they could be potential fertilizers that can provide nutrients for the improvement of soil physical and chemical properties.

No previous studies have been found regarding the use of hydrothermal synthetized LTA and FAU-X zeolites in their iron-modified forms for phosphate removal from aqueous medium. This work is devoted to the incorporation of iron (oxy)hydroxide on LTA and FAU-X zeolites for the improvement of their ability as phosphate carriers by promoting adsorption processes. Therefore, in this study we developed two novel adsorbents with the ability to recover phosphate efficiently at neutral pH range values (e.g., 6–8.5 natural pH values of treated urban wastewater), which do not require pH adjustment for full-scale application. A complete evaluation was performed to recover phosphate from synthetic wastewater, with its regeneration for reuse in sorption and desorption cycles for tertiary wastewater treatment purposes. Most studies regarding modified zeolites are focused on the removal mechanism, with little attention given to their possible regeneration. The final disposal of these hydrothermal synthetized zeolites modified with iron (LTA-Fe and FAU-X-Fe) as soil amendment materials is also considered in this work, since they do not represent a risk for water and soil environment due to lack of toxic elements that can be released from LTA-Fe and FAU-X-Fe to soils.

The objectives of this study are to (i) synthesize LTA and FAU-X zeolites by hydrothermal method, (ii) incorporate iron (oxy)hydroxide over synthetized zeolites, (iii) verify the influence of sorption parameters (pH, thermodynamic behavior, and concentration) for phosphate removal by LTA-Fe and FAU-X-Fe zeolites, (iv) determine the equilibrium and kinetic sorption parameters, and (v) validate the regeneration capacity of LTA-Fe and FAU-X-Fe zeolites.

## 2. Materials and Methods

### 2.1. Synthesis of Zeolite Samples

The zeolites Linde type A (LTA) and faujasite X (FAU-X) were obtained by the hydrothermal method reported by García et al. [18]. The sodium metasilicate nonahydrate (Na_2_O_3_Si∙9H_2_O) and commercial sodium aluminate solution (NaAlO_2_) were used as starting materials. The ratios and the composition of the synthesis mixtures used for the zeolites preparation are detailed in Table 1.

Firstly, the sodium metasilicate was dissolved in deionized water at room temperature. The measured amount of solution containing sodium aluminate was gradually poured into the solution containing sodium metasilicate with continuous stirring. The slurry was agitated for one hour at room temperature until a homogeneous gel was obtained. The gel was transferred to a closed plastic container and heated at 90 °C for the crystallization of LTA in the next four hours. In the case of the synthesis of FAU-X, once the homogeneous gel was obtained, the gel was subjected to 24 h aging period at room temperature. Then, the gel was transferred into a closed plastic container for the crystallization of FAU-X in the next eight hours with heating at 90 °C. The resultant LTA and FAU-X were vacuum-filtered and washed several times until the supernatant was at pH value 7. The obtained LTA and FAU-X were dried in an oven at 90 °C for further grind and storage.

### 2.2. Obtaining of Iron Form of Zeolites LTA and FAU-X

The iron form of zeolites (LTA-Fe) and (FAU-X-Fe) were obtained by treating a measured amount of each zeolite in 0.1 M of FeCl_3_ solution using a mass/volume ratio of 30 g/250 mL [1]. The mixture was stirred under reflux conditions (at 95 ± 2 °C) for 3 h. The pH (7) of the system was maintained by constant addition of NaOH solution. The procedure was repeated once under the same conditions, only refreshing the FeCl_3_ solution. The resultant LTA-Fe and FAU-X-Fe were washed with deionized water until no chloride ion was detected in the discharge. The zeolites in the iron form were dried in an oven and stored for further characterization.

### 2.3. Physicochemical Characterization

The specific surface area of the LTA and FAU-X zeolites on their parent and iron form LTA-Fe and FAU-X-Fe were determined by using the nitrogen gas adsorption single point method on an automatic sorption analyzer (Micrometrics Chemisorb 2720, Norcross, GA, US). A flow rate at 20 mL min^−1^ (standard conditions for temperature and pressure, STP) for a gas mixture containing 30% nitrogen (N_2_) diluted in helium (He) was used. The morphology surface and chemical composition of parent and iron form of zeolite samples were studied by a field emission scanning electron microscope at 20 kV (FE-SEM) (JEOL, Peabody, MA, USA JSM-7001F, Peabody, MA, USA). The chemical composition of the samples was determined by an energy-dispersive spectroscopy system (Oxford Instruments X-Max, Oxford, UK, Resolution 129 eV) coupled to the FSEM. Composition analyses reported are the average of at least four analyses for each material sample. The X-ray diffraction (XRD) patterns were acquired on a powder X-ray diffractometer (D8 Advance A25 Bruker, Karlsruhe, Germany) with a Cu Kα anode (λ = 0.1542 nm) operating at 40 kV and 40 mA. The diffraction patterns were collected at 25 °C and over an angular range from 4 to 60° of 2θ by a fast lineal LynxEyeXE detector (Bruker AXS, Karlsruhe, Germany). The infrared absorption spectra were recorded with a Fourier transform FTIR spectrometer (4100 Jasco, Easton, MD, USA) in the range of 4000–550 cm^−1^. KBr was used to prepare a table sample, and the spectra were obtained by collecting 32 scans using a 4 cm^−1^ resolution. The point of zero charge (PZC) of both iron forms of zeolite samples was determined by the pH drift method (ΔpH = pH_f_ - pH_i_ = 0), in the range of pH 2–11 as described in or previous work [19]. An amount of 0.1 g of zeolite was equilibrated with 25 mL of solution at three different ionic strengths (0.01, 0.05, and 0.1 M NaCl) at room temperature (22 ± 2°C).

### 2.4. Influence of the pH on Phosphate Adsorption

Synthetic wastewater solution was prepared from a NaH_2_PO_4_.2H_2_O stock solution (1000 mg L^−1^ PO_4_^3−^) in deionized water. A measured amount of zeolite (0.25 g LTA-Fe or FAU-X-Fe) was equilibrated in 25 mL of solution (25 mg L^−1^ PO_4_^3−^) at room temperature (22 ± 2 °C) at initial pH values of 3 to 10. The supernatant was separated by centrifugation at 5000 rpm and further filtration through 0.45 μm. The pH and phosphate concentration of the system were controlled at initial and equilibrium state. The tests were performed in batch by triplicate, and the average values are reported. The equilibrium adsorption capacity was calculated by Equation (1).

(1) $$q_{e}=\frac{v\times \left(c_{o}-c_{e}\right)}{w}$$

where *q_e_* is the equilibrium adsorption capacity (mg g^−1^ PO_4_^3−^), *v* is the volume of solution (L), *c_0_* and *c_e_* are the initial and equilibrium phosphate concentration (mg L^−1^ PO_4_^3−^), and *w* is the mass of the zeolite adsorbent (g).

The standard method was used for phosphate content determination [20]. The phosphate ion concentration was determined by the vanadomolybdophosphoric acid colorimetric method (4500-P C).

### 2.5. Equilibrium Phosphate Adsorption

Firstly, the enhancement of phosphate adsorption due to the incorporation of iron in both zeolites (LTA and FAU-X) was confirmed. A measured amount of zeolite (0.25 g of LTA, LTA-Fe, FAU-X, and FAU-X-Fe) was suspended in 25 mL of solution containing 25 mg L^−1^ PO_4_^3−^ at pH 7 at room temperature (22 ± 2 °C). Then, the equilibrium phosphate adsorption capacity was determined using a measured amount of zeolite (0.25 g LTA-Fe or FAU-X-Fe) equilibrated in 25 mL of solution containing 10–2000 mg L^−1^ PO_4_^3−^ at pH 7 (which is the condition of the treated wastewater). These assays were performed at three temperatures: 22 ± 2 °C (room temperature), 26 °C, and 30 °C. The supernatant was separated by centrifugation at 5000 rpm and further filtration through 0.45 μm. The pH and phosphate concentration of the system were controlled at initial and equilibrium state. The tests were performed in triplicate and the average values are reported. The equilibrium adsorption capacity was calculated by Equation (1).

### 2.6. Kinetic of Phosphate Adsorption

A measured amount of zeolite (0.25 g LTA-Fe or FAU-X-Fe) was suspended in 25 mL of solution containing 25 mg L^−1^ PO_4_^3−^ at pH 7 at room temperature (22 ± 2 °C). Samples (5 mL) were withdrawn at given times for controlling the phosphate concentrations and the pH in solution. The adsorption kinetic tests were performed in triplicate and the average values are reported. The phosphate adsorption capacity as function of time was calculated by Equation (2).

(2) $$q_{t}=\frac{v\times \left(c_{o}-c_{t}\right)}{w}$$

where *q_t_* is the equilibrium adsorption capacity (mg g^−1^ PO_4_^3−^), *v* is the volume of solution (L), *c_0_* and *c_t_* are the initial and phosphate concentration at specific time (mg L^−1^ PO_4_^3−^), and *w* is the mass of the zeolite adsorbent (g).

### 2.7. Phosphate Fractioning

The fraction of phosphate bonded to saturated zeolites (FAU-X-Fe and LTA-Fe) was determined based on a modified three-sequential-step extraction protocol [21]. The labile fraction, metal fraction, and the alkaline fractions were quantified. A measured amount of zeolite (0.25 g LTA-Fe or FAU-X-Fe) was suspended in 25 mL of solution containing 25 mg L^−1^ PO_4_^3−^ at pH 7 at room temperature (22 ± 2 °C). The solid phase was separated from the aqueous phase for further tests. The loosely bound phosphate fraction (physical-bound) was extracted from the saturated adsorbent sample (0.25 g), two consecutive times in 10 mL of 1 M NH_4_Cl (pH 7). The metal-bound fraction (e.g., iron, aluminum, etc.) was extracted two times in 10 mL of 0.1 M NaOH, followed by extraction in 1 M NaCl. The phosphate linked to the alkaline fraction (e.g., sodium, etc.) was extracted two times in 10 mL of 0.5 M HCl. Finally, the residual phosphate (other type of bound) was obtained by means of mass balance between the phosphate adsorbed and the extracted fractions.

### 2.8. Zeolites Regeneration

A measured amount of zeolite (0.25 g LTA-Fe or FAU-X-Fe) was suspended in 25 mL of solution containing 25 mg L^−1^ PO_4_^3−^ at pH 7 at room temperature (22 ± 2 °C). The solid phase was separated from the aqueous phase for further tests. Therefore, the loaded zeolite samples were equilibrated in aqueous solutions containing NaHCO_3_ (0.5 mol L^−1^ y pH 8.5). The pH and phosphate concentration of the regenerate solution was controlled at initial and equilibrium state. A second cycle of phosphate adsorption was performed to validate the regenerability of LTA-Fe or FAU-X-Fe. The tests were performed in triplicate and the average values are reported.

## 3. Results and Discussions

### 3.1. Zeolites Characterization

The process obtained 55 g LTA and 47 g FAU-X from 100 g of silicon source (Na_2_O_3_Si∙9H_2_O) used for the zeolites preparation. The specific surface area of LTA zeolite was 369 m^2^ g^−1^ and for FAU-X was 406 m^2^ g^−1^. The surface area values obtained by the LTA and FAU-X zeolites obtained in this study were in accordance with those reported by García et al., (2016) [18]. The synthetized LTA developed a high surface area, which is contradictory to previous works that reported very low values [22]. Instead, high surface area values have been reported for LTA due to the N_2_ access to some spaces in the mesoporous sections. This phenomenon has been reported to occur due to the loose packing of crystallites during crystallization. The larger crystallization times promoted the larger cubic crystals breaking down into smaller crystals [23]. However, the reduction of the surface area of LTA-Fe and FAU-X-Fe to 50 m^2^ g^−1^ and 60 m^2^ g^−1^ was determined after the iron hydroxide impregnation stage. The surface area values of the zeolite LTA-Fe and FAU-X-Fe decrease due to the appearance of extra framework cation size, as has been reported before. The decrease of the surface area of FAU-X was reported when larger-sized cations were introduced in the supercage of the zeolite, as occurred in this study [24].

**Figure 1:**
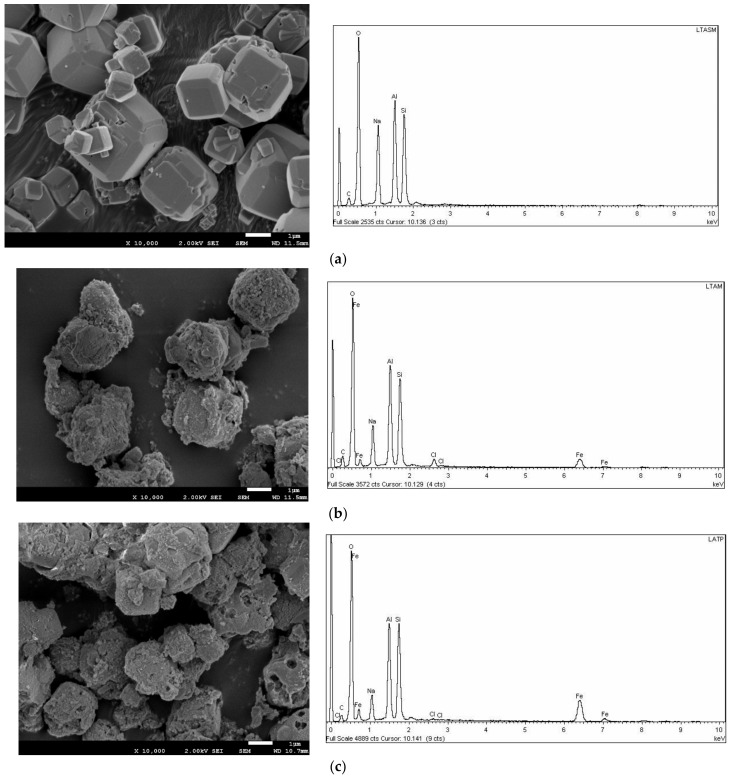
FSEM-EDX of the LTA zeolite: (**a**) parent form LTA, (**b**) iron form LTA-Fe, and (**c**) loaded phosphate.

**Figure 2:**
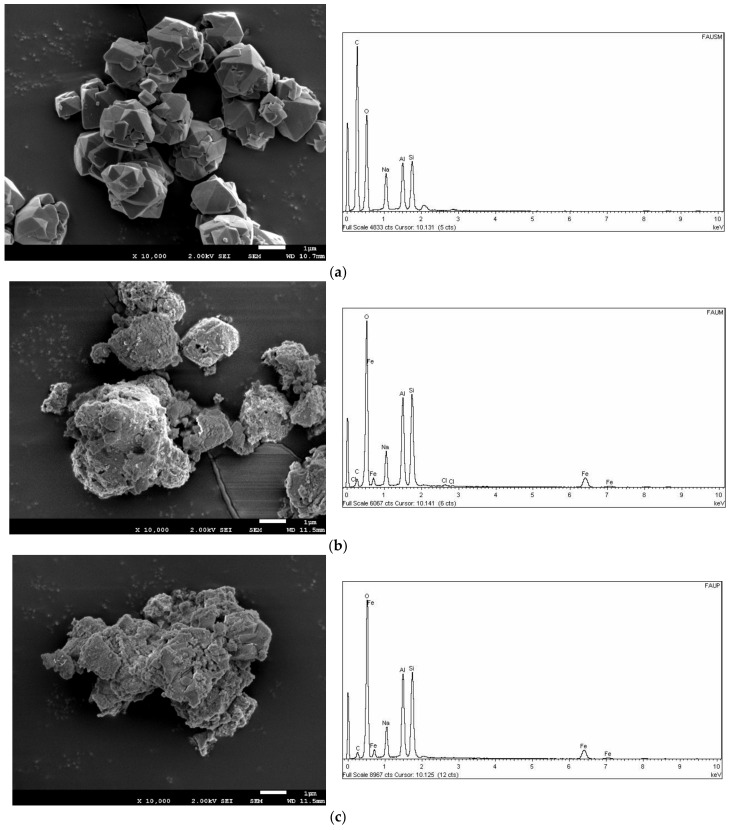
FSEM-EDX of the faujasite X zeolite: (**a**) parent form FAU-X, (**b**) iron form FAU-X-Fe, and (**c**) loaded phosphate.

The SEM images demonstrated the typical LTA zeolite morphology (Figure 1a–c). The exclusive existence of cubic crystals structures with well-defined edges characterized the synthesized LTA zeolite. A homogeneous particle size was not found; it ranged between 1 μm to 5 μm [14]. On the other hand, the SEM images demonstrated the typical faujasite type X (FAU-X) cubic crystals morphology (Figure 2a–c). The FAU-X zeolite was characterized by the unique existence of well-defined octahedral shape of particles. A heterogeneous particle size that ranged from 0.5 μm to 5 μm was also evidenced [25]. Both the LTA-Fe and FAU-X-Fe zeolites turned yellow after the iron impregnation as the main change. The morphology of the LTA-Fe and FAU-X-Fe zeolites evidenced the homogeneous loading of iron distributed over the zeolite framework. The surface of LTA-Fe and FAU-X-Fe appeared to be layered with particles covering the surface of parent LTA becoming rough, as has been reported before [26]. An enhancement of the roughness of zeolite surfaces occurred after phosphate adsorption onto the LTA-Fe and FAU-X-Fe.

The elemental compositions of both zeolites, Lynde type A and faujasite type X, are summarized in Table 2. Both LTA-Fe and FAU-X-Fe zeolites in the iron hydroxide form are characterized for containing iron and chloride after the modification. The results of the elemental composition of both LTA-Fe and FAU-X-Fe zeolites suggest the occurrence of ion exchange reactions between sodium and iron–chloride. The increase of iron–chloride content is characterized by the reduction of sodium content in both zeolites. The presence of Fe–Cl complexes (e.g., FeCl^+2^, FeCl_2_^+^) as predominant species of the solution (FeCl_3_) used for iron impregnation has been reported before [1]. In our previous work with a natural zeolite in the sodium form, the species distribution diagram and the exchange reactions were also proposed.

**Figure 3:**
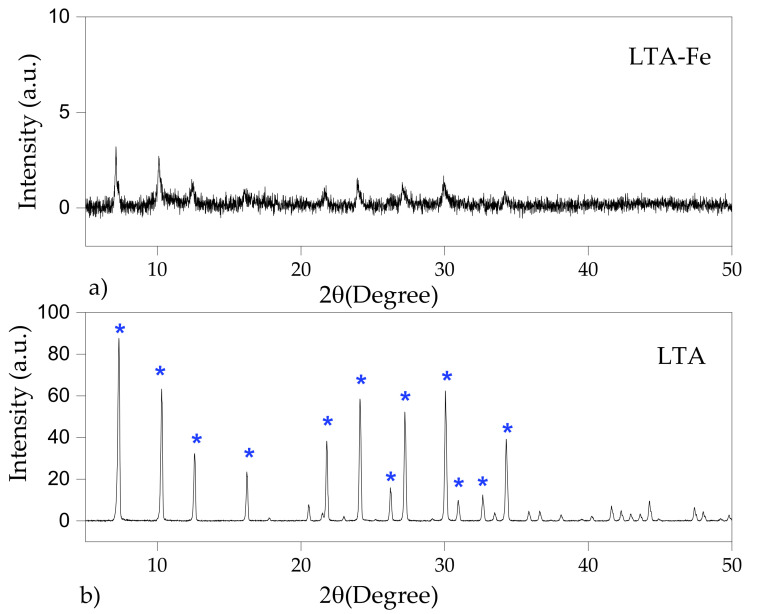
XRD of the LTA zeolite: (**a**) parent form LTA and (**b**) iron form LTA-Fe.

**Figure 4:**
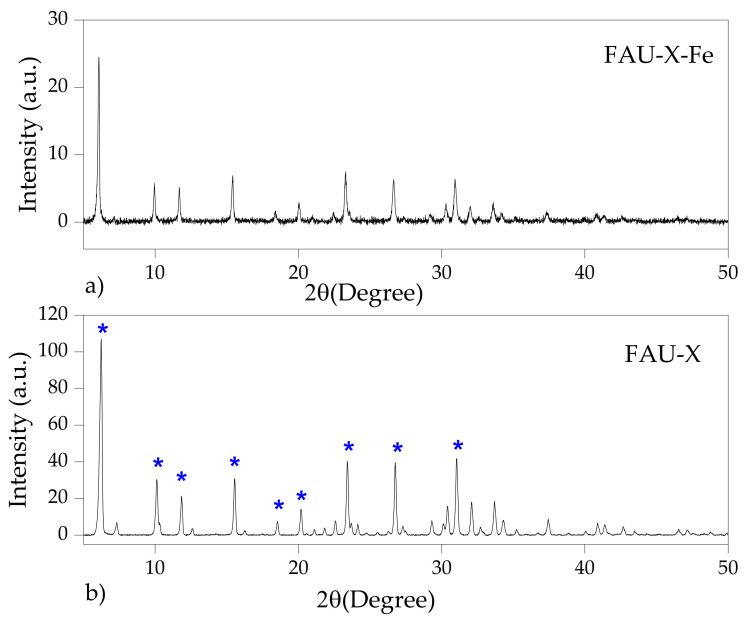
XRD of the FAU-X zeolite: (**a**) parent form FAU-X and (**b**) iron form FAU-X-Fe.

The XRD patterns of LTA and FAU-X zeolites are depicted in Figure 3 and Figure 4, respectively. The main reflection peaks of LTA zeolite were found at 2θ: 7.33° (100), 10.28° (110), 12.58° (111), 16.22° (210), 20.52° (211), 21.48° (220), 24.10° (310), 26.22° (222), 27.22° (320), 30.04° (400), 30.93° (410), 32.72° (331), and 34.28° (421) [27]. The presence of the characteristic reflections of the LTA zeolite were similar to the LTA standard reference of the International Zeolite Association [28]. The peaks are indexed in the cubic space group Fd-3m. The refined unit cell parameters are 11.90 Å of lattice. The characteristic reflection peaks of FAU-X zeolite were confirmed at 2θ: 6.15° (111), 7.31° (200), 10.33° (220), 11.83° (311), 15.53° (331), 18.52° (511), 20.24° (440), 22.57° (620), 23.67° (533), 26.74° (642), and 31.02° (751) [15]. The peaks are indexed in the cubic space group Fd-3m and the lattice parameter was calculated to be a = b = c = 24.95 Å. Then, the effective synthesis of highly crystalline Linde type A and faujasite type X zeolites was confirmed due to the well-defined and high intense XRD peaks in comparison to the reference pattern. The intensity of the strong and well-defined diffraction peaks of parent LTA-Fe zeolite as well as the FAU-X-Fe were drastically reduced, which is attributed to the amorphization of the crystal structure of the iron form of zeolite [26]. Conventionally, the Fe^3+^ incorporation occurred in the zeolite framework in tetrahedral or in the extra-framework octahedral sites [29]. Since the obtaining of LTA-Fe and FAU-X-Fe were performed at pH 7, the primary mechanism is the precipitation of iron hydroxides over the surface of zeolites. The existence of solids (iron hydroxides) in the LTA-Fe and FAU-X-Fe zeolites was confirmed through DRX analysis. The incorporation of iron into the zeolites structures is less likely to occur. However, in this study, the basal space d_100_ plane was calculated as 12.06 Å for the LTA at 2θ: 7.33, and it was compared with the d_001_ value for the LTA-Fe as 12.41 Å at 2θ: 7.11. The basal space d_111_ plane was calculated as 14.36 Å for the FAU-X at 2θ: 6.15, which was compared with the d_111_ value for the LTA-Fe as 14.58 Å at 2θ: 6.06. Then, the slight differences found in these crystallographic parameters suggest the partial incorporation of iron into the zeolites structure. Initially, Fe^3+^ reaches the extra-framework octahedral sites by diffusion, and after that, with the addition of NaOH, the Fe^3+^ is changed into the tetrahedral framework sites via isomorphic substitution, as has been reported when iron is incorporated in synthetic zeolites [29]. Additionally, higher amorphization of LTA zeolite was evidenced by the high reduction of the intensity of the peaks in comparison to the FAU-X. This fact can be explained in terms of the higher number of extra-framework sites of faujasite for Fe^3+^ in the cages that do not affect their structure [29]. No new crystalline iron phases were detected in the iron form of zeolites (LTA-Fe and FAU-X-Fe). Any new diffraction line appeared in the diffractogram of both zeolites, which can be attributed to the low contents which are below the limit of quantification of the XRD equipment.

**Figure 5:**
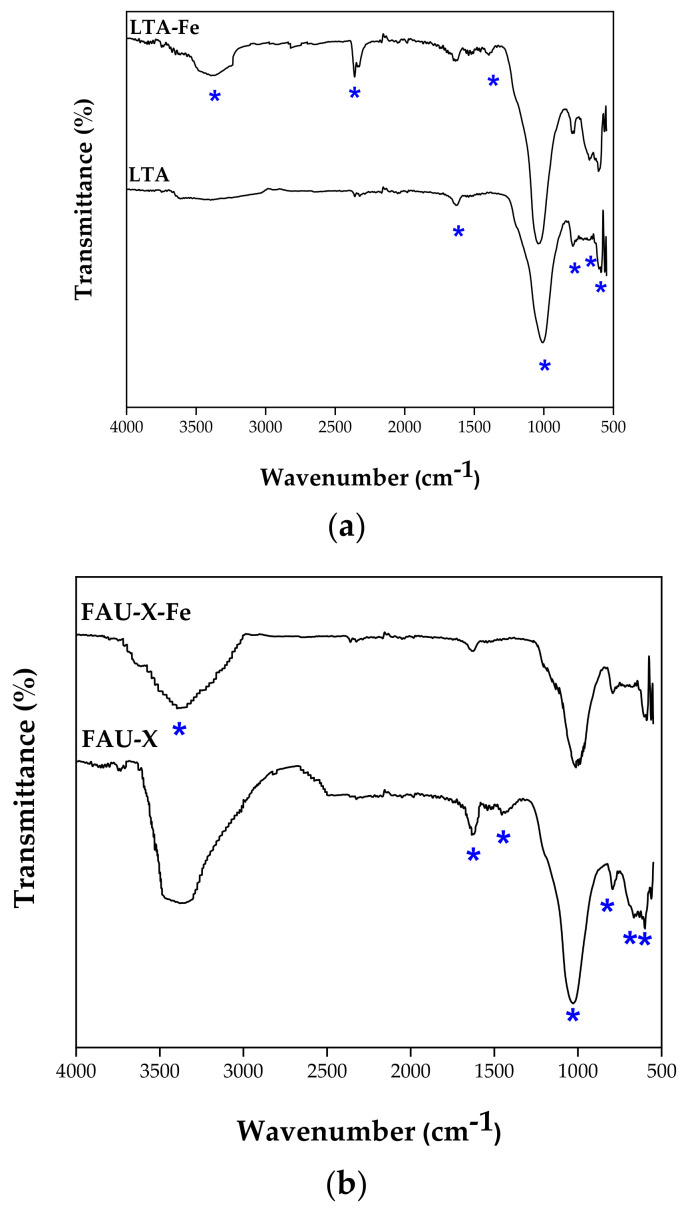
FTIR of the (**a**) LTA zeolite in parent and iron form LTA-Fe, (**b**) FAU-X zeolite in parent and iron form FAU-X-Fe.

The FTIR spectra of LTA and FAU-X zeolites are depicted in Figure 5a,b. The characteristic bands of both LTA (1007 cm^−1^ and 547 cm^−1^) and FAU-X (1011 cm^−1^ and 549 cm^−1^) zeolites were observed. These bands were attributed to LTA and FAU-X structure corresponding to the bending, symmetric stretching, and asymmetric stretching vibrations of internal tetrahedra, respectively. Particularly, in LTA, the absorption band at 560 cm^−1^ is attributed to the vibrations of sodalite cages that are linked by double four-rings (D4Rs). In FAU-X, the band at 760 cm^−1^ is assigned to the symmetric stretching vibrations of external linkages, while at 565 cm^−1^ it is connected with vibrations of sodalite cages, which are linked by double six-rings (D6Rs) [12]. Afterwards, the iron impregnation, the absorption bands described above changed, which can be attributed to the deterioration of the zeolite framework. The amorphization of both zeolites, LTA-Fe and FAU-X-Fe, is in concordance with the XRD results. The existence of Fe oxides in the hydrothermally synthetized zeolites was identified in the FTIR spectrum. There were determined changes in the absorption band at 567 cm^−1^ which represent the vibrations of Fe–O and Fe–O–Fe bonds [30]. Additionally, there are some bands between 3400 and 3600 cm^−1^ and the band at 1640 cm^−1^, which are assigned to the OH groups of adsorbed and zeolitic water, respectively. In the LTA-Fe zeolite, the appearance of new bands was evidenced at 2360 cm^−1^ and 1390 cm^−1^, which is in concordance with the incorporation of iron [26]. The shift of bands below the 1011 cm^−1^ and at near 1390 cm^−1^ occurred after iron impregnation. The shift of the bands described above are attributed to the breaking of Si–O bonds due to incorporation of Fe into the three-dimensional silica network [6]. As previously mentioned, Fe^3+^ reached the extra-framework octahedral sites and isomorphically substituted into the tetrahedral framework sites. The bands in the range of 3400 and 3600 cm^−1^ of LTA-Fe and FAU-X-Fe shifted to give a broad peak centered at 3300 cm^−1^, due to the interaction between OH groups and iron. Then, these new bands are related to the presence of iron hydroxide groups (≅FeOH). Thus, the formation of new functional groups (e.g., (>ZO^−^)_2_ (FeOH^2+^) or > ZO^−^ FeOH_2_^+^) occurred in the surface of zeolites [1], as was corroborated by SEM analysis.

### 3.2. Effect of pH on Phosphate Adsorption

**Figure 6:**
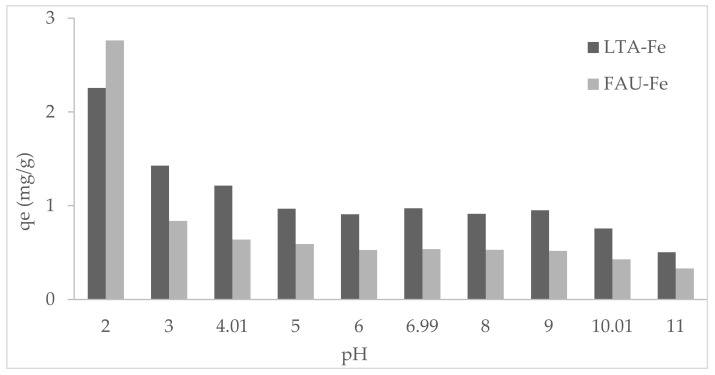
Phosphate adsorption (*q_e_*) as function of the initial pH solution pH (V: 25 mL, w: 0.25 g, and Ci: 25 mg PO_4_^3−^ L^−1^).

The phosphate adsorption onto LTA-Fe and FAU-X-Fe is depicted in Figure 6. The phosphate adsorption capacity onto LTA-Fe was higher than FAU-X-Fe within the range of pH 3 to pH 11. The highest phosphate adsorption capacity was reached at pH 2 and pH 3 for both iron zeolites. Then, the phosphate adsorption was also promoted by the form of orthophosphate (e.g., H_2_PO_4_^−^), even the orthophosphate acid is dominant (e.g., H_3_PO_4_). The phosphate adsorption capacity values were similarly in the range of pH value from pH 4 to pH 8. Instead, the reduction of the adsorption capacity values was evidenced in the range of pH values from pH 9 to pH 11. This behavior can be explained in terms of the pH of the solution due to the oxyanionic speciation of phosphate and the existence of positive and negatives charges according to the pH_PZC_ of the adsorbent. The point of zero charge was determined to be pH_PZC_: 7.8 ± 0.2 and 7.9 ± 0.3 for LTA-Fe and FAU-X-Fe, respectively. The point of zero charge was determined as the average of the plot lines at each ionic strength with ΔpH = 0, as they are depicted in Figure S1a,b. The orthophosphate species (e.g., H_2_PO_4_^−^ and HPO_4_^2−^) were involved in electrostatic attraction by positive charge due to the protonation of -(OH)^+^ groups over the zeolite surface below the pH_PZC_ [31]. The high basicity phosphate species (HPO_4_^2−^) has a pair of high electronic density that can form hydrogen bonds with the protonated LTA-Fe and FAU-X-Fe zeolite surface -(OH)^+^ groups (e.g., (>ZO^−^)_2_ (FeOH^2+^) or > ZO^−^ FeOH_2_^+^) [5]. On the other hand, above the pH_PZC_, the repulsive effect between orthophosphate species (mainly HPO_4_^2−^) and negative charge due to the hydroxylation occurred, as well as the hard Lewis base (OH^−^ ions) over zeolite [32]. The occurrence of electrostatic interactions (physisorption mechanisms) promotes outer-sphere adsorption complexes on the surface of LTA-Fe and FAU-X-Fe zeolites due to the protonation and hydroxylation reactions [6].

### 3.3. Phosphate Adsorption Isotherms: Thermodynamical Characterization

The phosphate adsorption on LTA-Fe and FAU-X-Fe and their parent forms are reported in Table 3.

The phosphate adsorption capacity values reached by LTA-Fe and FAU-X-Fe in comparison to their parent forms are three times and four times higher, respectively. Both synthetic zeolites are five times higher than 3.4 ± 0.2 mg PO_4_^3−^ g^−1^ reported for an iron-natural zeolite (clinoptilolite) used for phosphate removal [1].

The experimental equilibrium phosphate adsorption data were fitted to two isotherm models. The Langmuir model is represented in the linearized form by Equation (3).

(3) $$\frac{c_{e}}{q_{e}}=\frac{c_{e}}{q_{m}}+\frac{1}{k_{L}q_{m}}$$

where *q_m_* is the maximum adsorption capacity (mg PO_4_^3−^ g^−1^), and *k_L_* is Langmuir adsorption constant (L mg^−1^). In the Langmuir isotherm model, the favorability of the adsorption process is defined by the separation factor *r_L_* when 0 < r_L_< 1 and can be calculated by Equation (4). It is a dimensionless constant that explains the Langmuir isotherm shape.

(4) $$r_{L}=\frac{1}{1+k_{L}c_{0}}$$

The Freundlich model is represented in the linearized form by Equation (5).

(5) $$lnq_{e}=lnk_{F}+\frac{1}{n}lnc_{e}$$

where *k_F_* (mg g^−1^) is the maximum adsorption capacity (mg PO_4_^3−^ g^−1^) and *n* is the Freundlich constant.

The experimental data of phosphate adsorption capacities of LTA-Fe and FAU-X-Fe according to the Langmuir and Freundlich isotherms are summarized in Table 4. Similar phosphate adsorption capacity values of 18.5 and 17.5 mg PO_4_^3−^ g^−1^ were reached at pH 7 for LTA-Fe and FAU-X-Fe, respectively. The phosphate speciation in aqueous solution suggests the actuation of H_2_PO_4_^−^ and HPO_4_^2−^ anionic forms. At low phosphate concentration (until 200 mg L^−1^), the equilibrium data are well fitted to the Freundlich isotherm model for LTA-Fe and FAU-X-Fe zeolites. However, at overall equilibrium concentrations, R^2^ values of 0.95–0.97 for LTA-Fe and FAU-X-Fe were revealed. This fact corroborates the occurrence of physisorption (electrostatic interaction or outer-sphere complexation) as was well discussed above (Section 3.2). A second mechanism was established since the phosphate equilibrium adsorption data of LTA-Fe and FAU-X-Fe were best fitted to the Langmuir isotherm model (R^2^~0.99). In addition, the separation factor *r_L_* for both LTA-Fe and FAU-X-Fe zeolites was determined to be 0 < *r_L_*< 1, which is in accordance with a favorable chemical adsorption. Then, the chemisorption is attributed to the existence of specific and equivalent sites over the surface of LTA-Fe and FAU-X-Fe. The development of iron (oxy)hydroxides over the surface of both zeolitic structures endorse reactions incorporating covalent bonds. Thus, the phosphate adsorption is promoted by inner-sphere complexation through monodentate and bidentate complexes. Thus, the iron hydroxide structures complexed by surface hydroxyl groups are relevant groups for solutes removal [9]. Phosphate anions adsorption by iron (oxy)hydroxides supported on zeolites seems to be a practical solution for the potential use of this material at full scale due to the hydroxide’s particle size problem.

The thermodynamic studies allowed the prediction of adsorption mechanisms by chemical and physical interactions. The experimental data were fitted according to the thermodynamic laws’ parameters described by Gibbs free energy (Δ*G*^0^, kJ mol^−1^) description and determining the enthalpy (Δ*H^0^*, kJ mol^−1^) and entropy (Δ*S*^0^, kJ mol^−1^ K^−1^) values of the adsorption reactions by using Equations (6) and (7) [33].

(6) $$\Delta G^{0}=-RTlnk_{c}$$

The relationship between Δ*G*^0^, Δ*H*^0^, and Δ*S*^0^ is obtained as Equation (6), the well-known Van’t Hoff equation.

(7) $$lnk_{c}=\frac{-\Delta H^{0}}{R}\times \frac{1}{T}+\frac{\Delta S^{0}}{R}$$

where $k_{L}$ (L mg^−1^) is the Langmuir constant, which could be obtained as a dimensionless parameter. The $k_{c}$ is obtained as a dimensionless parameter by multiplying *k_L_* by the molecular weight of adsorbate (*M_w_*, g mol^−1^) and then by factors 1000 and 55.5, which is the number of moles of pure water per liter, described in Equation (8) [34]. R is the universal gas constant (8.314 J mol^−1^ K^−1^) and T is the absolute temperature (K).

(8) $$k_{c}=k_{L}\times M_{w}\times 1000\times 55.5$$

The values of thermodynamic parameters (e.g., Δ*G*^0^, Δ*S*^0^, and Δ*H*^0^) of the phosphate adsorption are summarized in Table 5. The positive values of enthalpy (Δ*H*^0^), 39.80 and 24.01 kJ mol^−1^ for the phosphate adsorption on LTA-Fe and FAU-X-Fe, respectively, demonstrate that the process was endothermic. However, the negative values of Gibbs free energy (Δ*G*^0^) for both zeolites are associated with the spontaneous decrease of energy at higher temperatures. Additionally, positive values of (Δ*S*^0^), 0.18 and 0.15 kJ mol^−1^ K^−1^ for LTA-Fe and FAU-X-Fe, respectively, are attributed to the increase of the disorder at the interface of the solid-solutions system [35]. Additional information is brought by the Δ*G*^0^ values, which demonstrate that complexation reactions for phosphate adsorption are the main mechanisms followed by the influence of electrostatic interaction [36].

### 3.4. Kinetic of Phosphate Adsorption Processes

**Figure 7:**
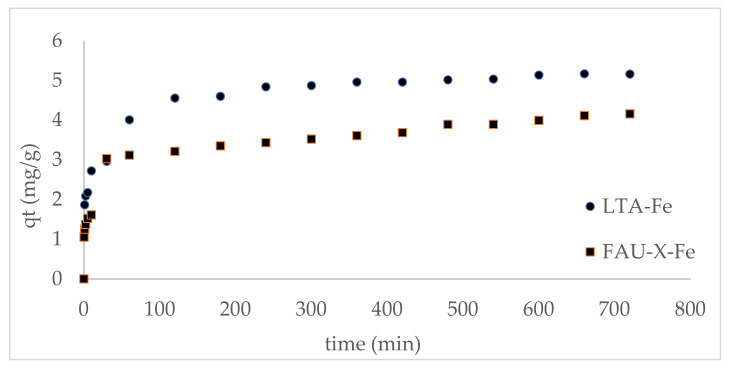
Kinetic profile of phosphate adsorption onto LTA-Fe and FAU-X-Fe (V: 25 mL, w: 0.25 g, and Ci: 25 mg PO_4_^3−^ L^−1^).

The phosphate adsorption capacity by LTA-Fe and FAU-X-Fe as a function of time is represented in Figure 7. For FAUX-Fe, 80% of the total equilibrium attainment was achieved in less than 10 min, while for LTA-Fe, 50 min were required to achieve this threshold. These times to reach equilibrium attainment are compatible with applications in stirred tank reactors. The fast initial stage seems to be governed by physisorption due to the hydrogen bond interaction, as was discussed above. In the slow adsorption stage, the chemisorption occurred due to the higher time and energy requirements [6].

The experimental data of phosphate equilibrium sorption kinetics were fitted to the kinetics model of pseudo-first-order (Equation (9)), pseudo-second-order (Equation (10)), and intraparticle diffusion model (Equation (11)) that considered that adsorption might be influenced by diffusion in the spherical adsorbent and by convective diffusion in the phosphate solution.

(9) $$ln\left(q_{e}-q_{t}\right)=ln\left(q_{e}\right)-k_{1}t$$

(10) $$\frac{t}{q_{t}}=\frac{1}{k_{2}q_{e}^{2}}+\frac{t}{q_{e}}$$

where $k_{1}$ (h^−1^) and $k_{2}$ (g mg^−1^ h^−1^) are the kinetics constants.

(11) $$q_{t}=k_{t}t^{\frac{1}{2}}+A$$

where $k_{t}$ (mg g^−1^ h^−1/2^) is the intraparticle diffusion rate constant and A (mg g^−1^) is a constant that provides information about the thickness of the boundary layer. The homogenous particle diffusion model was computed for the phosphate sorption onto LTA-Fe and FAU-X-Fe. If diffusion occurred in the film phase ($D_{f}$, m^2^ s^−1^), the adsorption rate is described by Equation (12), but when the rate of adsorption is controlled by LTA-Fe and FAU-X-Fe particle diffusion ($D_{p}$, m^2^ s^−1^), it can be determined by Equation (13) [37].

(12) $$-ln\left(1-\left(\frac{q_{t}}{q_{e}}\right)\right)=\frac{D_{f}C_{s}}{hrC_{z}}t$$

(13) $$-ln\left(1-{\left(\frac{q_{t}}{q_{e}}\right)}^{2}\right)=\frac{2\pi^{2}D_{p}}{r^{2}}t$$

where *C*_s_ (mg L^−1^) and *C*_z_ (mg kg^−1^) are the phosphate concentrations in solution and in the LTA-Fe and FAU-X-Fe zeolites, respectively, *r* is the average radius of the LTA-Fe and FAU-X-Fe zeolites particles (particles below 200 mesh ≈ radius: 3.7 × 10^−5^ m), t is the contact time (min), and h is the film thickness of the LTA-Fe and FAU-X-Fe particle (1 × 10^−5^ m for a poorly stirred solution).

The kinetic parameters of phosphate adsorption by LTA-Fe and FAU-X-Fe are summarized in Table 6. The kinetic adsorption data were best fitted to the pseudo-second-order model in accordance with an R^2^ value ≈ 0.99. The phosphate adsorption is promoted by the monodentate and bidentate complexation, as was discussed above. Additionally, important information was provided by the intraparticle diffusion model because the existence of three stages of adsorption was verified. Then, a multi-stage adsorption governed the phosphate adsorption onto LTA-Fe and FAU-X-Fe. The fast initial adsorption stage occurred by the film diffusion of phosphate through the hydrodynamic layer and further diffusion through the boundary layer to the external surface of the zeolites. The secondadsorption stage is attributed to the intraparticle diffusion process that governed the phosphate adsorption at a slowed-down rate. Finally, the last stage is characterized by the simultaneous reduction of phosphate content in synthetic wastewater and the saturation of active sites of LTA-Fe and FAU-X-Fe that promoted the equilibrium state [36].

### 3.5. Phosphate Fractioning

The fraction values of the chemical forms of phosphate that are bound by adsorption to LTA-Fe and FAU-X-Fe zeolites are summarized in Table 7. The phosphorous fraction immobilized by physisorption was about 28.6% and 33.2% for LTA-Fe and FAU-X-Fe, respectively. This fraction corresponds to the loosely bound phosphate fraction (LB-P), which is available for plants. The second fraction is the one where phosphate is bonded to metallic species of iron in their (oxy)hydroxide form due to chemisorption. The phosphate fractions bonded to iron (oxy)hydroxides of LTA-Fe and FAU-X-Fe are 64% and 59%, respectively. The important incidence of chemisorption followed by physisorption for phosphate bonding to LTA-Fe and FAU-X-Fe was discussed above and again confirmed. In addition, the phosphate bounded to sodium fraction turned out to be minimal; average values of 5% and 6% were determined, confirming the relevance of iron complexed by surface hydroxyl groups, which are relevant groups for phosphate removal in comparison to other functional groups (e.g., sodium hydroxyl groups). Finally, a residual phosphate fraction by means of mass balance was obtained. Residual phosphate fractions of 3% and 2% were bonded to LTA-Fe and FAU-X-Fe. No comparable information has been found regarding phosphate fractioning synthetized zeolites LTA and FAU-X. Comparable results were reported for the phosphate bonded to iron metal fraction for natural zeolite and synthetic zeolites (LTA-Fe and FAU-X-Fe). However, the phosphate bonded too loosely, and sodium fraction values resulted as different for natural zeolite and synthetic zeolites. Since the chemical composition of most of the natural zeolite includes the presence of calcium and magnesium, both cations contribute to the phosphate immobilization by chemical precipitation, implying the formation of low-solubility calcium and magnesium phosphates [1].

### 3.6. Phosphate Desorption Processes

**Figure 8:**
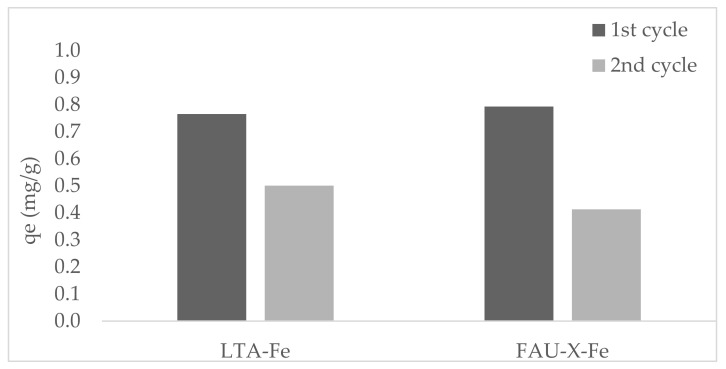
Phosphate capacity in two continuous adsorption-desorption cycles for LTA-Fe and FAU-X-Fe (V: 25 mL, w: 0.25 g, and C_i_: 25 mg PO_4_^3−^ L^−1^).

The phosphate adsorption capacity of LTA-Fe and FAU-X-Fe after being regenerated is represented in Figure 8. An average of 30% was recovered from the saturated LTA-Fe and FAU-X-Fe, which correspond to the loosely bond phosphate. Thus, the physisorbed phosphate fraction was easily removed from saturated LTA-Fe and FAU-X-Fe zeolites.

The remaining phosphate over LTA-Fe and FAU-X-Fe zeolites was not recovered due to the strong bonding through chemical complexes to iron (oxy)hydroxides of zeolites. In the second cycle, a reduction of the phosphate adsorption capacity occurred at 30% and 50% for LTA-Fe and FAU-X-Fe, respectively. The occupancy of bonding sites blocked the adsorption of new phosphate species. Additionally, during regeneration at pH 8.5, both orthophosphate species (e.g., H_2_PO_4_^−^ and HPO_4_^2−^) were released from saturated LTA-Fe and FAU-X-Fe. The LTA-Fe and FAU-X-Fe have a limited reusability with a reduction of efficiency after each cycle of adsorption–desorption. Thus, the immobilized phosphate through chemical bonds, which is the main mechanism of adsorption, cannot be desorbed. However, the saturated LTA-Fe and FAU-X-Fe can be lastly disposed for soil amendment.

### 3.7. Advantages and Disadvantages of Phosphate Adsorption Using Hydrothermally Synthetized LTA-Fe and FAU-X-Fe Zeolites

The phosphate adsorption capacities from this study were compared to the values reported for other inorganic materials, summarized in Table 8. There are many adsorbents developed for phosphate removal (e.g., natural zeolites, synthetic zeolites, and natural clays). Conversely, most of the studies are focused on the removal mechanism with little attention to their possible regeneration or the final disposal. Nevertheless, the economic and regulatory concerns about the promotion of phosphorous recovery and valorization strategies are scarce. In the European Union, a new Directive on Fertilizers would be useful to encourage the quality control of byproducts to classify the type of agronomic applications or potential applications in the soil amendment [6].

As was reported in our previous works with natural zeolites, they are very selective for phosphate removal. The enrichment of trace levels of hazardous metals was determined when urban wastewater was used. Hence, they do not represent concerns about accumulation and further release from the sorbent to the soil. In this case, the use of two hydrothermally synthetized zeolites modified with iron was evaluated for phosphate removal. The hydrothermal method was selected since it is a cheaper and more straightforward method in comparison to others. In addition, the hydrothermal method does not require specialized equipment, and has low energy requirements. The use of water as part of the synthesis process makes it environmentally friendly. Additionally, the rejection of hazardous components from the zeolite structure occurred during crystallization due to the selectivity of the process for the crystal growth.

The phosphate adsorption capacities of our synthetic zeolites (LTA-Fe and FAU-X-Fe), which are around six-fold in comparison to the modified natural zeolites, are a remarkable finding of this study. Additionally, LTA-Fe and FAU-X-Fe were around eight-fold in comparison to the synthetic zeolite obtained from fly ashes. The phosphate adsorption capacities of LTA-Fe and FAU-X-Fe were comparable with some other iron oxide supports and particles. However, a critical stage of all adsorbents is the final disposal, which must be environmentally friendly. In our case, the synthetic zeolites (LTA-Fe and FAU-X-Fe) developed limited reusability. The great advantage of LTA-Fe and FAU-X-Fe zeolites based on our previous studies is the opportunity of being reused for soil amendment application as slow nutrient release and for plants growth. In comparison, other types of adsorbents, such as synthetic zeolites obtained from fly ashes, may contain hazardous pollutants.

In summary, there are many advantages and limitations in the use of LTA-Fe and FAU-X-Fe. However, the purpose of this work is to provide information about the use of hydrothermally synthetized LTA-Fe and FAU-X-for phosphate recovery purposes, simultaneously being an alternative for wastewater treatment technology.

## 4. Conclusions

In this study, two zeolites types, LTA and FAU-X, were synthetized by the hydrothermal method. Both parent zeolites were enriched with iron (oxy)hydroxides, obtaining their iron forms LTA-Fe and FAU-X-Fe for the phosphate recovery from synthetic wastewater. A good efficiency for phosphate adsorption was developed by LTA-Fe and FAU-X-Fe at pH around 7, which is the real condition of treated wastewater. The development of adsorbents with this characteristic supposed an improvement in comparison with other conventional materials used for phosphate adsorption. In fact, phosphate adsorption on LTA-Fe and FAU-X-Fe zeolites is higher than their parent forms and reached values of five times the adsorption of a natural zeolite. Physical and chemical adsorption described the phosphate removal by LTA-Fe and FAU-X-Fe. The hydrogen bonding (outer-sphere complexes) and complexation reactions (inner-sphere complexes) governed the phosphate adsorption onto LTA-Fe and FAU-X-Fe. Additionally, the phosphate adsorption process was mainly characterized by its spontaneity and endothermic behavior. The phosphate adsorption occurred faster in LTA-Fe than in FAU-X-Fe and was best explained by the intraparticular diffusion process. The speciation of phosphate in saturated zeolites was well explained in that chemical adsorption is the main mechanism of adsorption, followed by physical adsorption as a contributive route. The limited reusability of LTA-Fe and FAU-X-Fe was determined in two cycles of continuous operation which supposed a weakness in comparison to polymeric exchangers. However, the saturated phosphate solutions obtained from adsorbent regeneration can be used for soil amendment application. In addition, the saturated LTA-Fe and FAU-X-Fe could also be finally disposed of as alternative sources of phosphorous for soil amendment, since they do not contain or release hazardous contaminants from the adsorbent.

## Figures and Tables

**Table 1:** Synthesis conditions for the preparation of zeolites LTA and FAU-X.

Zeolite	SiO_2_/Al_2_O_3_	Na_2_O/SiO_2_	H_2_O/Na_2_O	Na_2_O_3_Si∙9H_2_O ^a^ (g)	NaAlO_2_^b^ (g)	H_2_O ^c^ (g)
LTA	2	3	40	50	179	310
FAU-X	4	2	46	56	99	222

Weight used of commercial starting materials: ^a^ sodium metasilicate nonahydrate; ^b^ sodium aluminate solution, and ^c^ deionized water.

**Table 2:** Elemental composition of zeolites: LTA, LTA-Fe, FAU-X, and FAU-X-Fe.

Zeolite	O (%)	Na (%)	Al (%)	Si (%)	Cl (%)	Fe (%)
LTA	58.9	14.1	13.3	13.7	<lq *	<lq *
LTA-Fe	60.9	7.7	12.3	12.4	1.3	5.3
FAU-X	58.7	14.8	12.3	14.2	<lq *	<lq *
FAU-X-Fe	60.7	7.3	11.6	13.9	0.3	6.2

* Below the limit of quantification.

**Table 3:** Comparative phosphate adsorption on LTA-Fe and FAU-X-Fe and their parent forms.

Zeolite	*q_e_* * (mg.g^−1^)
LTA	0.3
LTA-Fe	0.9
FAU-X	0.2
FAU-X-Fe	0.8

* Parameters obtained at V: 25 mL, w: 0.25 g, and C_i_: 25 mg PO_4_^3−^ L^−1^.

**Table 4:** Phosphate adsorption isotherm parameters for LTA-Fe and FAU-X-Fe.

Zeolite	Langmuir *			Freundlich *		
	$q_{m}$ (mg.g^−1^)	$k_{L}$ (L.mg^−1^)	R^2^	$k_{F}$ (mg.g^−1^)	1/n	R^2^
LTA-Fe	18.5	0.007	0.99	0.37	0.59	0.97
FAU-X-Fe	17.5	0.006	0.99	0.41	0.53	0.95

* Parameters obtained at V: 25 mL, w: 0.25 g, and C_i_: 10–2000 mg PO_4_^3−^ L^−1^.

**Table 5:** Phosphate thermodynamic parameters for LTA-Fe and FAU-X-Fe.

Zeolite	Temperature (K)	ln $k_{c}$	R^2^	Δ*G*^0^ * (kJ mol^−1^)	Δ*S*^0^ * (kJ mol^−1^ K^−1^)	Δ*H*^0^ * (kJ mol^−1^)
LTA-Fe	315.15	6.44	0.95	−4.88	0.18	39.80
	319.15	6.70		−5.05		
	323.15	6.81		−5.16		
FAU-X-Fe	315.15	8.81	0.97	−5.70	0.15	24.01
	319.15	8.89		−5.80		
	323.15	9.04		−5.91		

* Parameters obtained at V: 25 mL, w: 0.25 g, and C_i_: 25 mg PO_4_^3−^ L^−1^.

**Table 6:** Kinetic parameters of phosphate adsorption for LTA-Fe and FAU-X-Fe.

Kinetic Model	Kinetic Parameter *	LTA-Fe	FAU-X-Fe
Pseudo-first-order	*q_e_* (mg g^−1^)	3.51	2.89
	k_1_ (h^−1^)	0.12	0.13
	R^2^	0.85	0.92
Pseudo-second-order	*q_e_* (mg g^−1^)	5.76	4.61
	*k*_2_ (g mg^−1^ h^−1^)	0.030	0.047
	R^2^	0.99	0.99
Intraparticle diffusion	*k_t_*_1_ (mg g^−1^ h^−1/2^)	2.64	3.39
	R^2^	0.84	0.90
	*k_t_*_2_ (mg g^−1^ h^−1/2^)	5 × 10^−1^	2 × 10^−1^
	R^2^	0.90	0.88
	*k_t3_* (mg g^−1^ h^−1/2^)	6 × 10^−1^	5 × 10^−1^
	R^2^	0.93	0.99
HPDF film diffusion	*D_f_* (m^2^ s^−1^)	9.27 × 10^−11^	2.45 × 10^−15^
	R^2^	0.96	0.95
HPDM particle diffusion	*D_p_* (m^2^ s^−1^)	2.42 × 10^−15^	2.11 × 10^−15^
	R^2^	0.95	0.97

* Parameters obtained at V: 25 mL, w: 0.25 g, and C_i_: 25 mg PO_4_^3−^ L^−1^.

**Table 7:** Fractions of phosphate bonded to LTA-Fe and FAU-X-Fe.

Zeolite	*q_e_* * (mg·g^−1^)	LB-P *		(Fe-Al)-P *		(Na)-P *		R-P *	
		(mg·g^−1^)	%	(mg·g^−1^)	%	(mg·g^−1^)	%	(mg·g^−1^)	%
LTA-Fe	15.6 ± 0.4	4.5 ± 0.2	28	10.0 ± 0.1	64	0.8 ± 0.1	5	0.4 ± 0.0	3
FAU-X-Fe	12.1 ± 0.6	4.0 ± 0.1	33	7.1 ± 0.2	59	0.7 ± 0.1	6	0.3 ± 0.0	2

* Values obtained at V: 25 mL, w: 0.25 g, and C_i_: 25 mg PO_4_^3−^ L^−1^.

**Table 8:** Summary of phosphate adsorption capacities of inorganic adsorbents.

Adsorbent	Description		Isotherm Models				Kinetic Models				
			Langmuir		Freundlich		Pseudo-First-Order		Pseudo-Second-Order		
			$q_{m}$	$k_{L}$	1/n	$k_{F}$	$k_{1}$	R^2^	$k_{2}$	R^2^	Ref.
			(mg g^−1^)	(L mg^−1^)		(m g^−1^)	(min^−1^)		g mg^−1^ min^−1^		
Synthetic zeolites	Hydrothermally synthetized	LTA-Fe	18.5	0.007	0.6	0.4	0.12	0.85	0.03	0.99	This study
		FAU-X-Fe	17.5	0.006	0.5	0.4	0.13	0.92	0.04	0.99	
Natural zeolites	Natural clinoptilolite	ZN	0.6	0.01	0.47	0.02	-	-	-	-	[9]
		Z-Al	7.0	0.02	0.32	0.85	0.2	0.93	0.6	0.9	
		Z-Fe	3.4	0.02	0.25	0.59	0.1	0.92	0.2	0.99	[1]
		Z-Mn	5.6	0.01	0.34	0.95	-	-	-	-	[38]
Synthetic zeolite	From fly ash with lanthanum	LMZ	2.31	3.09	0.59	1.54	-	-	-	-	[39]
Natural clays	Natural form	C_1_	21.4	0.0018	0.7	0.1	0.33	0.94	0.14	0.99	[6]
		C_2_	20.9	0.0098	0.8	0.1	0.15	0.79	0.22	0.97	
	Modified form	C_1_-Fe	38.0	0.0018	0.6	0.3	0.09	0.73	0.01	1.00	
		C_2_-Fe	37.6	0.0012	0.6	0.3	0.15	0.77	0.06	0.99	
Modified bentonite	Zn-containing bentonite clay		4.12	1.1	0.96	2.2	-	-	-	>0.99	[40]
	Pillared bentonite by Fe		11.15	0.6	0.81	4.4	-	-	-	>0.99	
Natural clays	Bentonite from Iran		0.369	0.01	0.58	12.85	-	-	-	-	[41]
	Zeolite from Iran		0.627	0.007	0.64	12.63	-	-	-	-	
	Kaolinite from Iran		0.624	0.005	0.62	11.94	-	-	-	-	
Modified bentonite	Pillared bentonite by Fe/Al		8.33	0.03	0.26	0.18	-	-	-	-	[42]
Na-Bentonites	Pillared bentonite with Al		12.7	1.61	0.22	7.56	-	-	-	1	[43]
	Pillared bentonite with Fe		11.2	1.83	0.16	7.43	-	-	-	0.99	
	Pillared bentonite with Fe-Al		10.5	1.25	0.21	5.54	-	-	-	1	
Metals-modified bentonite clay	Bentonite (Bent) modified with Fe, Co and Ni	Fe-Bent	20.88	0.111	0.11	9.86	0.0090	0.956	0.0040	0.996	[44]
		Co-Bent	46.95	0.648	0.12	23.03	0.0020	0.928	0.0034	0.981	
		Ni-Bent	29.07	0.496	0.11	13.44	0.0070	0.963	0.0091	0.965	
		Bent	6.57	0.281	0.15	2.44	0.0023	0.927	0.024	0.998	
Iron oxide/hydroxide nanoparticles-based agglomerates	Iron nanoparticles	AggFe	122.0	-	-	-	-	-	-	-	[45]
Iron (oxyhydr)oxides	Ferrihydrite	Fh	57	-	-	-	-	-	-	-	[46]
	Goethite	Gt	9.5	-	-	-	-	-	-	-	
	Hematite	Hm	4.75	-	-	-	-	-	-	-	

## Supplementary Materials

The following supporting information can be downloaded at https://www.mdpi.com/article/10.3390/ma15155418/s1: Figure S1: Diagrams of initial pH vs. ΔpH for determination of point of zero charge of the LTA zeolite: (a) LTA-Fe and (b) FAU-X-Fe.
