# Supplementary material for: LTA and FAU-X Iron-Enriched Zeolites: Use for Phosphate Removal from Aqueous Medium
Source: Materials (Basel). 2022 Aug 5;15(15):5418. doi: 10.3390/ma15155418 (PMC9369556; doi:10.3390/ma15155418)
Supplement: Supplementary file 1 [file materials-15-05418-s001.zip › materials-1806909-supplementary.pdf]

LTA and FAU-X iron-enriched zeolites: use of for phosphate removal from aqueous medium

Diana Guaya [1,2*], Hernán Cobos [1], Jhulissa Camacho [1], Carmen Milena López[1], César Valderrama [2,3] and José Luis Cortina [2,3]

[1] Department of Chemistry, Universidad Técnica Particular de Loja, Loja, Ecuador
[2] Department of Chemical Engineering, BarcelonaTECH-UPC, Barcelona, Spain
[3] Barcelona Research Center for Multiscale Science and Engineering, 08930 Barcelona, Spain

* Correspondence: deguaya@utpl.edu.ec

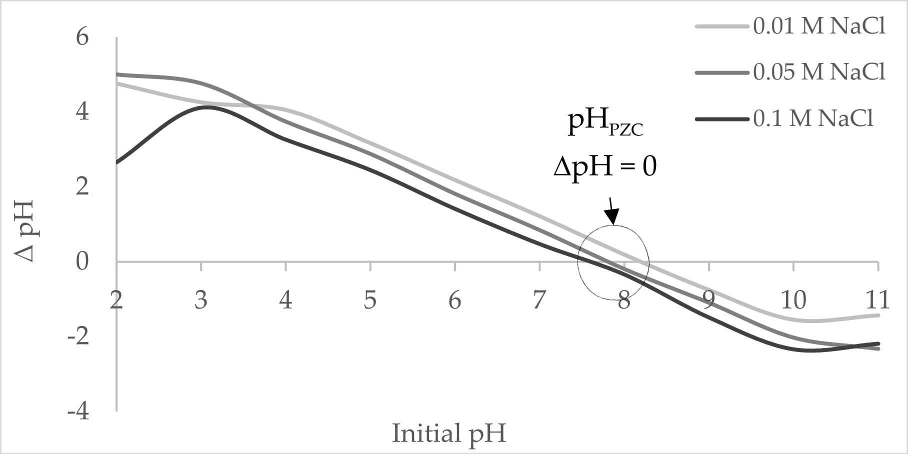

(a)

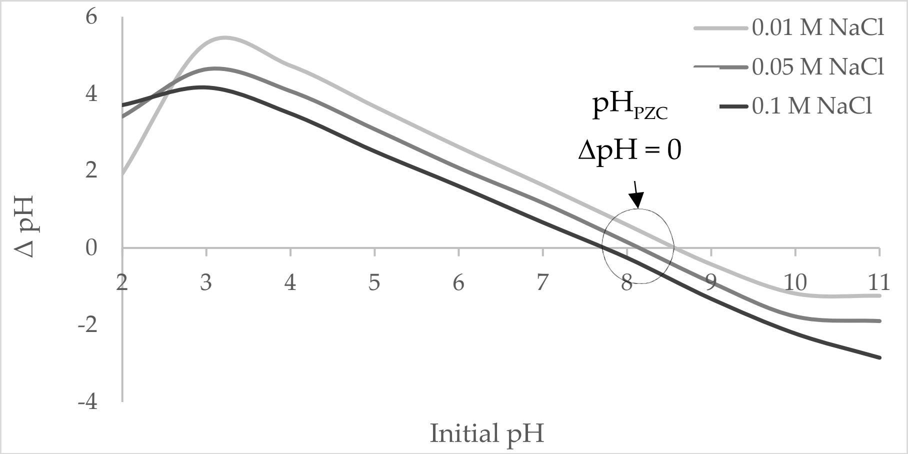

(b)

Figure S1. Diagrams of initial pH vs ΔpH for determination of point of zero charge of the LTA zeolite: (a) LTA-Fe and (b) FAU-X-Fe.
